# User Perspectives on a Resilience-Building App (JoyPop): Qualitative Study

**DOI:** 10.2196/28677

**Published:** 2021-07-08

**Authors:** Aislin R Mushquash, Erin S Pearson, Kayla Waddington, Angela MacIsaac, Shakira Mohammed, Elizabeth Grassia, Savanah Smith, Christine Wekerle

**Affiliations:** 1 Department of Psychology Lakehead University Thunder Bay, ON Canada; 2 School of Kinesiology Lakehead University Thunder Bay, ON Canada; 3 Department of Pediatrics McMaster University Hamilton, ON Canada

**Keywords:** resilience, smartphone, app, innovation, qualitative, perspective, mHealth, emotion, mental health

## Abstract

**Background:**

Resilience is the capability, resources, and processes that are available to a person or system to adapt successfully in the face of stress or adversity. Given that resilience can be enhanced, using advances in technology to deliver and evaluate the impact of resilience interventions is warranted. Evidence supports the effectiveness of the resilience-building JoyPop app in improving resilience-related outcomes after use; however, experiential data from users is also needed to provide a more comprehensive account of its utility.

**Objective:**

The aim of this study was to explore users’ experiences with the JoyPop app and their perspectives on its utility.

**Methods:**

This qualitative description study involved a combination of group and one-on-one semistructured interviews with a subset of first-year undergraduate students who participated in a larger evaluation of the JoyPop app. Participants used the app for a 4-week period and were subsequently asked about their frequency of app use, most and least used features (and associated reasons), most and least helpful features (and associated reasons), barriers to use, facilitators of use and continuation, and recommendations for improvement. Data were coded and categorized through inductive content analysis.

**Results:**

The sample of 30 participants included 24 females and 6 males, with a mean age of 18.77 years (SD 2.30). App use ranged from 1 to 5 times daily (mean 2.11, SD 0.74), with the majority indicating that they used the app at least twice daily. The Rate My Mood, Journal, and SquareMoves features were reported to be used most often, while the Rate My Mood, Journal, and Breathing Exercises features were identified as the most helpful. A number of themes and subthemes pertaining to facilitators of app use (prompts, creating routine, self-monitoring opportunities, expressive opportunities), barriers to app use (editing, lack of variety, student lifestyle), outcomes of app use (increased awareness, checking in with oneself, helpful distraction, emotional control), and recommendations for app improvement (adding more features, enhancing existing features, enhancing tracking abilities, providing personalization) were identified.

**Conclusions:**

This study provides insight into the aspects of the JoyPop app that motivated and benefitted users, as well as measures that can be taken to improve user experiences and promote longer-term uptake. Users were willing to engage with the app and incorporate it into their routine, and they valued the ability to self-monitor, express emotion, and engage in distraction.

## Introduction

### Background

Resilience is the multifaceted capability of an organism or system to adapt to challenging circumstances [1,2]. As adaptive creatures, humans must continually cope with stressors as they arise and find ways to flourish despite setbacks. A large part of this ability to cope and adapt involves the use of foundational skills that underlie resilience, including self-regulation processes such as emotion regulation [3-7].

Because resilience is developed and shaped over time, the argument follows that resilience can be promoted [8]. It is beneficial to assist individuals during moderately stressful experiences by providing them with self-regulatory skills (eg, emotion regulation) when they need them so that these skills might be added to their coping repertoire over time and become habitual resilient responses in times of stress [8]. The objective of this study was to explore user experiences with a resilience-building intervention (the JoyPop app [9,10]) and their perspectives on its utility.

### Building Resilience

Mobile health (mHealth) app use has greatly expanded in recent years, with the benefit of reaching a wider population than traditional health interventions [11] and providing greater ease of access [12,13]. Recent evidence has demonstrated that mHealth interventions, including apps, are feasible and effective methods of increasing symptom reporting and improving medication and treatment adherence and health knowledge in those with chronic health conditions [14-16]. Evidence to date has also supported an mHealth approach for many different mental health–related targets, such as mood disorders, stress, and substance use [17,18], with emerging support for mHealth approaches to resilience [9]. Focusing on building resilience through mHealth approaches is in line with the recent recommendations that mental health-related apps be geared toward general well-being and fostering self-regulatory skills (eg, emotion regulation) as opposed to focused on addressing specific mental health disorders [19].

### Evaluating mHealth Apps

Although there is promise in promoting resilience with apps, evaluating an app’s utility requires a multimethod approach that includes a quantitative examination of changes seen in target outcomes following app use, along with qualitative data exploring users’ perspectives on the app and its utility. Although establishing the effectiveness of an app via quantitative data is important and often lacking [20], doing so does not ensure that an app will be used or accepted by the target audience [21]. Interestingly, the alignment of an app with evidence-based interventions is often unrelated to its popularity [22], and some of the more popular apps contain little evidence-based content [23]. Obtaining user perspectives can provide insight into patterns of use and continuation over time [18,24-26].

User acceptance and uptake of an app have often been measured through proxy metrics, such as utilization data and download rates; however, qualitative data can better reveal the features and functions that draw users in and keep them engaged [27,28]. This is made especially clear by the evident eagerness of app users to communicate their desires and preferences. For example, app reviews posted to popular web-based stores (eg, Google Play Store, Apple App Store) often contain requests for certain features and design changes [25,27]. Obtaining user input throughout the design and evolution process helps to ensure that the needs of the target audience are met and would likely increase the possibility of uptake [25,27]. Gleaning perspectives from users is also in line with a proposed Canadian assessment framework for e–mental health apps, which indicates that apps should be user-centered and user-desirable such that they reflect the needs and expectations of potential users [29].

### Evaluation of the JoyPop App

**Figure 1:**
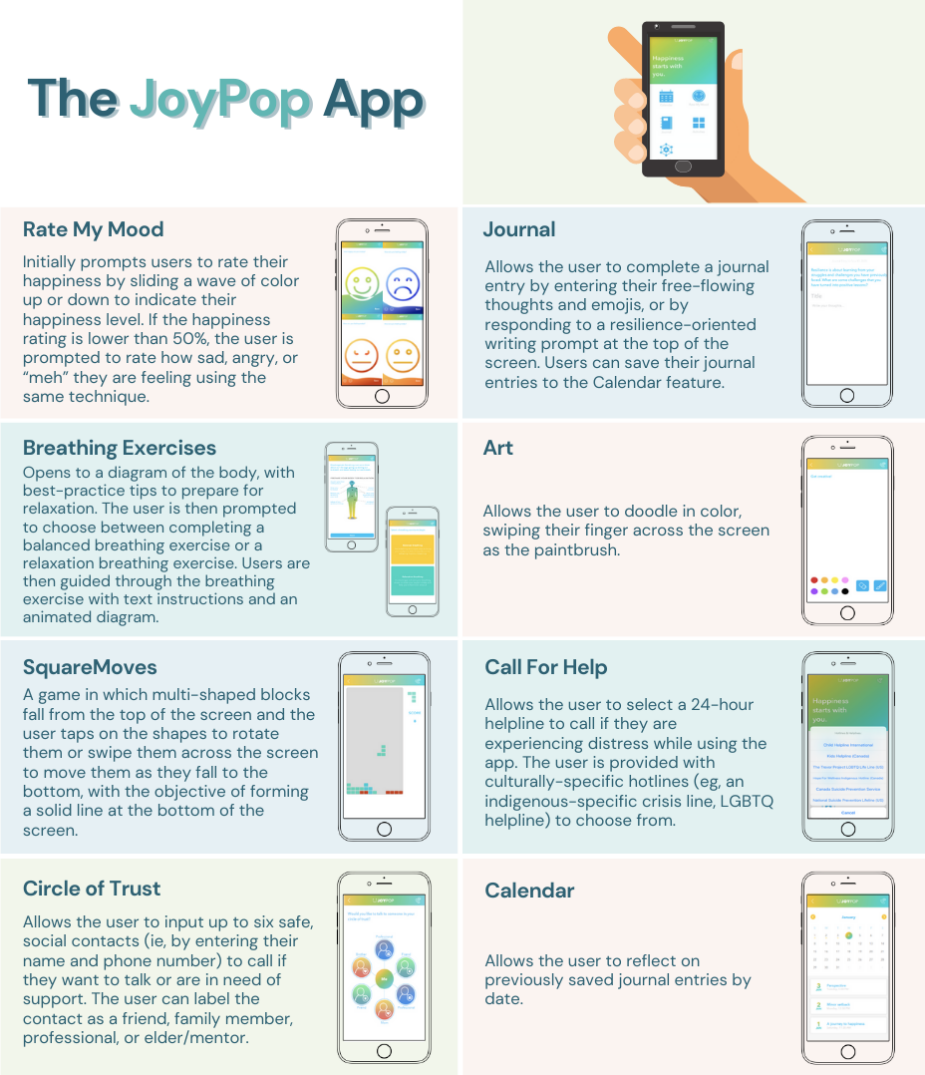
Features of the JoyPop app. LGBTQ: lesbian, gay, bisexual, transgender, queer.

The resilience-building JoyPop app (see Figure 1) targets self-regulation through features that promote self-reflection and self-awareness [10]. As documented by MacIsaac et al [9], the JoyPop app was developed to focus on daily self-regulation through evidence-based techniques. Rate My Mood is a self-monitoring feature meant to increase insight and regulation of emotions [30,31]. The Breathing Exercises are intended to decrease arousal in support of increased self-regulation [32]. The Journal feature encourages self-reflection and includes a positive focus that is supported by research [33,34]. SquareMoves is a Tetris-like game that is included for its ability to induce a “flow”-like state in which the user is completely involved in the activity, thereby providing a positive distraction and decreased negative self-focus [35]. The Art feature allows users to express their creativity through doodling, which can be used as a nonverbal emotional outlet [36]. Social connection to one’s support network is provided with the Circle of Trust feature, which allows users to input contact information and quickly reach out to their supports when help or connection is needed. Direct access to established helplines is also provided through the app; this function has been identified as important to mHealth app users [19,25,27,37].

The JoyPop app was designed with youth, who have demonstrated a willingness to use mHealth apps [28,38], as a target audience. Research conducted by a federally funded Canadian team examining pathways to resilience for youth who had experienced adversity informed early decisions on feature inclusion [39-43]. In addition, consultation with youth, service providers, and clinician-scientists informed initial discussions with app developers at Clearbridge Mobile (Wekerle and Smith 2021, unpublished data). Once an initial version of the app was developed, youth involved with child welfare and victim services as well as providers who worked with youth provided input that was used to revise the features and design. Additional information on JoyPop is available on the web [10].

Research to date suggests that the JoyPop app contributes to positive changes in outcomes related to resilience among youth. In a sample of undergraduate students making the transition to university who were asked to use the app for 4 weeks, improvements in emotion regulation and depression symptoms were evidenced with each additional day a participant used the app [9]. Further, improvements in emotion regulation were especially evident for individuals who had experienced a greater degree of adversity during childhood. While the transition to university is associated with new and exciting opportunities, it is also marked by stress and an increased prevalence of mental health difficulties [44,45], making it an important time to support resilience and adaptive coping skills.

Gathering qualitative evidence on users’ experiences with the app and perspectives of its utility is important for a comprehensive evaluation. For instance, because the JoyPop app contains numerous features that might differentially contribute to changes in outcomes, a qualitative assessment could elucidate which features and functions participants felt were most used and most helpful. Moreover, with the goal of promoting autonomous use of the app outside of a research context, a qualitative assessment would help gather feedback on factors facilitating continued long-term use.

### Objective

The objective of this study was to describe users’ experiences with the JoyPop app and their perspectives on its utility. A priori hypotheses were not specified due to the nature of the qualitative research design.

## Methods

### Design

This study was part of the larger program of research examining the outcomes associated with using the JoyPop app [9]. Qualitative description, a naturalistic inquiry approach, was used as an overarching framework, as this method is most appropriate when a straightforward description of a phenomenon is sought [46,47]. Ethical approval was received from Lakehead University’s Research Ethics Board prior to commencing the study.

### Participants

Undergraduate students who were enrolled in their first year at a mid-size Canadian university (ie, Lakehead University), were fluent in English, and had an iPhone were eligible for the larger study. As documented elsewhere [9], participants attended 3 group laboratory sessions: pre-app, mid-app (after 2 weeks of app use), and post-app (after 4 weeks of app use). During these sessions, participants received information about the JoyPop app, were provided with support in accessing the app, and completed self-report measures assessing quantitative outcomes. Participants were asked to use the JoyPop app at least twice per day over the 4-week study period; no additional requirements were made with respect to feature use or time spent using the app. Each morning and evening (ie, twice per day), participants were sent reminder emails to use the app.

Prior to their post-app session, participants were presented with the option of sharing their experiences using the app and perspectives on its utility by participating in this qualitative description study. Those who expressed interest were invited to participate and received CAD $10 (US $8.13) upon completion.

### Data Collection

Data were collected from February to March 2019 through a combination of group and one-on-one semistructured interviews immediately following participants’ completion of their self-report measures during the post-app laboratory session. Because the nature of each laboratory session was group based, this same format was selected for the interviews in an effort to maximize involvement while minimizing scheduling challenges and participant burden. This structure also enabled questions to be asked systematically in a direct manner by the interviewer to each participant on an individual level (in contrast with a focus group, where participants interact more with one another) [48-50]. One-on-one interviews were conducted when only 1 participant among those attending the post-app session expressed interest in participating or when a participant wished to participate but had a scheduling conflict with the group-based session. All interviews were conducted in a private board room on the Lakehead University campus.

The interview guide (see Multimedia Appendix 1), created for the purposes of the study, asked participants about their frequency of app use, most and least used features (and associated reasons), most and least helpful features (and associated reasons), barriers to use, facilitators of initial and continued use, and recommendations for improvement. The same interview guide was used during all group and one-on-one interviews. Interviewers (AM and SM) were enrolled in a master’s degree program in clinical psychology at Lakehead University with training and supervision related to interviewing and qualitative data collection. Interviewers were supervised by ARM, who is a Registered Clinical Psychologist with extensive training in interviewing and experience conducting qualitative research. Interviewers expanded beyond the interview guide (ie, probes and prompts) throughout each interview to promote elaboration on given responses. To ensure that input was received from all participants, interviewers sought to encourage responses through direct questioning, provision of elongated pauses, and reflective listening [46]. Each interview was audio recorded and transcribed verbatim.

### Data Analysis

All transcripts were deidentified and analyzed by ESP and KW independently. The data analysts first read and reread the transcripts to become familiar with the data. Inductive content analysis, whereby themes are not predetermined but emerge organically, was then used to code and categorize the text [51]. This involved the application of open coding (ie, writing detailed notes, headings, keywords, and commonly used phrases while reading). A list of reoccurring categories was then created and grouped into higher-order themes [46,51]. Following independent analysis, the data analysts met on multiple occasions and used an iterative, constant comparative method to discuss their findings and agree upon the organizing thematic framework. When differing interpretations occurred, the researchers continued discussions until consensus was reached [52]. Following this manual analysis phase, the transcripts were imported into NVivo Software 12 (QSR International) to verify the consistency and hierarchy of the resultant themes. Finally, the thematic framework was reviewed and discussed by all members of the researcher team to ensure consensus and that interpretations aligned with participants’ meanings and experiences [46,53]. Frequencies were applied to provide an account of overall app and specific feature use.

To enhance the trustworthiness of the data and limit researcher biases [46,54,55], several strategies were integrated into the data collection and analysis phases. For example, to promote credibility, member checking was done between questions to ensure the interviewers understood participant responses correctly [53]. The researchers also kept detailed notes throughout the duration of the study in the form of an audit trail to enable a transparent account of the data analysis process [46]. In addition, to enhance the dependability of the data and endorse neutrality, the analysis was conducted by two independent researchers (ESP, KW) who were trained in qualitative methodologies but removed from the conceptualization and data collection phases of the study.

## Results

### Participant Demographics

A sample of 30 first-year undergraduate students was enrolled in the study. Participants were predominantly female (24/30, 80%) and 18.77 years old on average (SD 2.30). Their ages ranged from 16-29 years, with the majority of the sample being aged 19 years or younger (28/30, 93%). Most participants self-identified as White (21/30, 70%), Asian (6/30, 20%), or Black (2/30, 7%). Close to half (14/30, 47%) of the participants were pursuing degrees within the area of health and behavioral sciences (eg, kinesiology, psychology, nursing), while 5/30 (17%) were undeclared/undecided. The remaining participants were pursuing degrees in science and environmental studies (7/30, 23%; eg, biology, geography), business administration (2/30, 7%; eg, business), or social sciences and humanities (2/30, 7%; eg, social work, political science). We conducted 8 group-based (3 with n=2; 4 with n=3; 1 with n=5) and 7 one-on-one interviews. On average, the group interviews were 16.19 minutes long, and the one-on-one interviews were 8.79 minutes long.

### Overall Use

At the outset of each interview, participants were asked how often they used the JoyPop app over the preceding 4 weeks. Use ranged from 1 to 5 times daily (mean 2.11, SD 0.74), with the majority indicating that they used the app at least twice daily. Some participants specifically reported that they used it first thing in the morning and again in the evening before going to sleep.

### Feature Use and Perceived Helpfulness

A summary of the use of specific features and their perceived helpfulness is presented in Table 1. Most participants indicated that the Rate My Mood, Journal, or SquareMoves features were used most often. Most participants identified either the Rate My Mood, Journal, or Breathing Exercises as most helpful. The Art feature was rated as the least used and reported most often as being the least helpful.

**Table 1:** Feature use and helpfulness (N=30).

Feature	Value, n (%)^a^			
	Most used	Least used	Most helpful	Least helpful
Rate My Mood	16 (53)	0 (0)	8 (27)	3 (10)
Journal	15 (50)	8 (27)	8 (27)	5 (17)
SquareMoves	16 (53)	5 (17)	3 (10)	3 (10)
Breathing Exercises	9 (30)	5 (17)	10 (33)	1 (3)
Art	2 (7)	13 (43)	0 (0)	8 (27)
Helplines	0 (0)	1 (3)	0 (0)	2 (7)
Circle of Trust	0 (0)	12 (40)	0 (0)	4 (13)

^a^Some participants identified >1 feature when asked about the most/least used and most/least helpful features.

### User Perspectives

In addition to summarizing the reported use and helpfulness, analyses of the transcripts revealed a number of themes and subthemes pertaining to facilitators of app use, barriers to app use, outcomes of app use, and recommendations for app improvement. Each theme and its associated subthemes are described below along with illustrative quotes.

### Facilitators of App Use

Participants described several positive attributes of the JoyPop app that facilitated their enjoyment and/or continued use. The most commonly discussed attributes included prompts; creating routine; self-monitoring opportunities; and expressive opportunities.

#### Prompts

Many participants noted their appreciation for the two types of prompts associated with the study design or app itself. Specifically, receiving email reminders to use the app and the prompts built into the Journal feature (ie, meant to provide suggestions for topics to write about) were identified as helpful.

I like to write and like, get my expressions out. But sometimes I like, struggle to start. So, having the prompts was nice...

Participant 4

At first, I felt like I didn’t really need the app…when I got the reminder and I kept on using it, I felt like it was actually really helpful.

Participant 27

#### Creating Routine

Participants discussed how over time, they developed a routine with using the JoyPop app. Many shared how they used it in the morning to start their day and in the evening to “decompress” and process the day’s events. Several participants also noted that the more they used the app, the easier and more enjoyable it became.

At first, it was just like “Oh, I have to use the app.” … And after a while, I just found myself just kind of using it on my own, not really thinking about having to do it, but wanting to do it.

Participant 8

Yeah it definitely got better with time. And like, incorporating the breathing exercises into like, your everyday routine. I found it really helpful.

Participant 10

#### Self-Monitoring Opportunities

Many participants commented about appreciating the ability to look back at their calendar and journal entries over time, which enabled them to review and reflect on previous feelings and circumstances. This act of self-monitoring was deemed to be helpful in gaining perspective on one’s feelings and current state and also facilitated continued use of the JoyPop app. Some participants also reported using various app features in tandem, such as journaling following their mood rating, which likely contributed to ongoing self-monitoring.

Look[ing] back, even if I was like, having a bad day I’d be like, “Oh, I was really happy on this day.” And if like, I’m feeling bad one day I could look back and see like, “Oh, this week I was really, really happy” kind of thing.

Participant 5

I liked being able to rate my mood and then go into the journal setting and be able to like, write down things that were happening that day, so I could go and look back.

Participant 13

#### Expressive Opportunities

Some participants shared how using the JoyPop app enabled them to express themselves and that this was a positive feature that encouraged future use. Some noted prior difficulty communicating their feelings verbally and stated that through the app, they were able to express their emotions more readily via the Journal or Rate My Mood features.

It helps give students like, the opportunity to have a way to express themselves... the journal [feature] for example. But also, like keeping track of how they’re feeling and keeping themselves in check by having like, that visual… showing them where they’re at with their emotions.

Participant 4

[I found that journaling] was really helpful at the end of the day, just to kind of unload everything…[it] unpacked my day so I found that was really helpful.

Participant 18

### Barriers to App Use

In addition to factors that facilitated enjoyment and ongoing use of the JoyPop app, participants noted some factors that they felt interfered with their use. The primary barriers were related to editing, lack of variety, and student lifestyle.

#### Editing

Some participants discussed the inability to make changes to their data in some of the features. For example, a few expressed that they did not feel comfortable writing in the Journal if they were not able to later edit or retract their entry. Similar sentiments were expressed by some participants regarding not being able to save their drawings in the Art feature. Participants expressed that the option to edit or save their data would have motivated them to use certain features more often.

I didn’t like how you couldn’t edit [the journal entries] because, [I’m] someone who can get anxious about making mistakes…

Participant 1

It didn’t save my mood and stuff, you know?… I also didn’t like that I couldn’t save my drawings. Like, or attach a drawing to a mood or something like that.

Participant 17

#### Lack of Variety

Some participants commented on the limited prompt variety in the Journal feature, indicating that once they had responded to all the available prompts, they felt less inclined to continue to use the feature. Some participants similarly noted that because the Rate My Mood feature included a limited number of emotions, they felt that their true feelings may not have been captured.

I think the quotes being the same every time when you fill out the [journal] got kind of [frustrating].

Participant 1

I liked the rate my mood the least… it was just like, a happy or sad thing. Would’ve been nice be able to have something that it’s like, there’s different moods to rate. So like maybe I was feeling very happy but there would have been one to track, like how nervous I was feeling or how distressed like I feel. Like, more options would have been nice.

Participant 3

#### Student Lifestyle

Some participants commented that, at times, the busy lifestyle and fatigue associated with being a student interfered with using the app. These participants indicated that “life” affected their use of the JoyPop app, and they mentioned feeling that there was not enough time to use it due to competing priorities and responsibilities.

[If] I was like, really busy studying, like during midterms [I would use it less].

Participant 3

If I was busy at the moment…if I was working on schoolwork or something…it wouldn’t happen.

Participant 28

### Outcomes Related to App Use

During the interviews, participants expressed several positive outcomes related to their use of the JoyPop app. These were categorized into four main subthemes: increased awareness; checking in with oneself; helpful distraction; and emotional control*.* While most participants noted positive outcomes, a few mentioned experiencing increased stress while using the app.

#### Increased Awareness

Many participants expressed how the JoyPop app enhanced their self-awareness in terms of being better able to recognize and differentiate their emotions. In addition, a few participants shared that after using the app, they began thinking more about the “why” behind their emotions, and how their feelings were related to mental and physical health.

It kinda gave me some time to reflect back on…certain things…After a point, I started noticing a pattern of why this one emotion I kept linking with a certain event, and then it kinda made me internalize like, “Am I depending too much on this event to make me feel a certain way?”

Participant 6

I was really impressed with the fact that it brought my attention more to how I was feeling. So, instead of just acknowledging that I was stressed and then managing with that, it really brought attention to why I was feeling that and just acknowledging that, which I thought was really cool.

Participant 18

#### Checking in With Oneself

The notion of taking or making time to check in with oneself was discussed frequently. In particular, participants mentioned benefits associated with pausing and reflecting on how they were feeling and what they might need in the moment (eg, to focus on their breathing). Related to their increased awareness, participants also expressed that because they were more aware of how they were feeling, they were able to “reset” and get things “back in check.”

It’s just nice to start your day off or end your day off with…taking a moment for yourself and like…getting your breathing in check and your emotions back in check.

Participant 4

[It] kind of like, forced me to take time throughout the day to reflect upon how I was feeling.

Participant 8

#### Helpful Distraction

Many participants expressed that some features of the JoyPop app helped them to “take their mind off of” negative feelings or stressors they were experiencing. The distraction provided by the Journal and SquareMoves were deemed particularly useful and enabled the participants to focus on “something else.”

I liked the journal one the most I think, ’cause it was like, different. It was kind of like, distracting to take your mind off of whatever you might have been feeling at the time.

Participant 5

There were times when I’d be like on my phone and like, bored and stressed out, and just looking for a distraction and then I was like, “Ok, this was something I could do right now.”

Participant 11

I think the [SquareMoves feature] was probably the most helpful in terms of like, turning off my thoughts and focusing on something else.

Participant 22

#### Emotional Control

Related to their increased awareness, many participants shared how the JoyPop app helped them to have more active control over their emotions. Using specific features (eg, Journal, Breathing Exercises) was discussed often as a means of managing emotions directly, especially in relation to school-specific situations (eg, midterm examinations, studying).

Take a couple more minutes and like, go through the events of my day… I kinda found that…as a good way to kinda, balance my emotions.

Participant 6

It really helps to calm down: the breathing exercises. It really helps like when I’m…angry… it helped a lot.

Participant 16

It definitely helped me figure [my feelings] out… actually helped me to relate my mood… Often I would feel better afterwards because I’d be like, “Ok. I know how to deal with this now”… rather than just sitting there and stewing not knowing what to do.

Participant 28

#### Increased Stress

A few participants believed that the app or specific features of the app contributed to increased stress. For example, one participant explained that they felt there were too many buttons popping up which distracted them while using the app.

Too many buttons to hit or same quotes popping up or it was just…Even though it wasn’t the app causing the stress, I found that in this situation it couldn’t get me out of it… [It] was too many buttons and distracting colors to help.

Participant 1

[Using the breathing feature] …I feel stressed and stuff when I use this.

Participant 18

### Recommendations for App Improvements

Suggestions for improving the JoyPop app involved four main subthemes: adding more features; enhancing existing features; enhancing tracking abilities; and providing personalization.

#### Adding More Features

Many participants suggested adding more features for future app users. Specifically, participants recommended adding more activities (eg, games and varied breathing exercises or strategies for relaxation). The notion of incorporating accountability was also discussed by a few participants, with suggestions to increase social connectedness through the app and adding a reward system tied to use patterns. Finally, a few participants felt that reminders sent through their phone (eg, via pop-up notifications) would lead to increased app use.

Some apps use like, this notification. Like, they notify you after not using [it] for an hour. So, maybe adding [more notifications on the app].

Participant 26

I think having more activities on it…or being able to connect with people through the app rather than having to call them…getting their opinion on coping strategies, or… maybe having them to… check in on you every once in a while… like have accountability.

Participant 28

#### Enhancing Existing Features

Some participants noted that more mood options would be beneficial in the Rate My Mood feature. Because only four emotions were included, the participants felt that their true feelings may not have been captured. Another common suggestion by participants was to include more prompts in the Journal feature. One participant suggested adding prompts to the Art feature as well.

[Adding] sound for the breathing so I can like, close my eyes or not like, stare down at my phone.

Participant 2

I like the idea of like the prompts for drawing or like a coloring book idea.

Participant 18

Um, definitely the rate the mood thing: adding more moods so then people can kinda have more options.

P23

#### Enhancing Tracking Abilities

Having the ability to track emotions over time was also discussed by several participants. For instance, participants suggested that having a chart or graph within the app would be helpful to look back at and compare their emotions over time.

Some means of actually being able to properly like… track all of my moods and how I’m feeling… it would be a good way of like, expressing myself and then being able to reflect on it if I wanted to.

Participant 3

#### Providing Personalization

A few participants suggested offering users the ability to personalize the app based on their personality or preferences. Others thought personalization based on locations might be beneficial.

Having different versions of it for your personality types. Like certain things that are geared more towards your personality than others… there are a lot more creative and artistic people that would gear from the creative parts, but then there are the people who are less creative that will not use it at all or can’t draw. So…different versions of it too for each personality type would definitely… help it.

Participant 21

Like, you could put your location in [the app] and then it will bring up the local places around you [helplines, resources].

Participant 23

## Discussion

### Principal Findings

Consistent with recent recommendations to evaluate the utility and usability of mHealth apps [18,23-25], this study sought to obtain user perspectives on the JoyPop app. Overall, we found that different features of the app varied in their uptake and perceived benefit to users. The app seemed to remind participants to take a moment for themselves to self-monitor and express their emotions, distract themselves from the stress of daily life, and gain emotional awareness and control strategies in the process. The establishment of routine, potentially via prompts, also appeared to facilitate app use. At the same time, certain feature limitations and a busy lifestyle seemed to dissuade use, and for a few participants, the app was perceived to add to their stress. Participants were eager to share many recommendations for feature additions and changes. Complementary to previous research on the effectiveness of the JoyPop app [9], the current study speaks to its usability and functionality and aspects that can be improved in this regard, as both effectiveness and usability are vital to establishing the merit of an mHealth app.

Certain aspects of participants’ experience with the JoyPop app emerged as key facilitators of continued use. First, one major subtheme was the creation of routine, whereby the drive to use the app seemed to progress from wanting to adhere to study requirements to eventually desiring to use the JoyPop app and feeling it had become a habitual part of one’s day. This idea of habit formation is intriguing, as it is consistent with prior work on the factors associated with technology uptake [56,57]. Habit formation constitutes a process by which app use comes to be associated with certain environmental or contextual cues, and such cues may trigger the behavior in the future with some degree of automaticity [56]. Accordingly, one feature of the study that may have encouraged habit formation was the inclusion of consistently timed email prompts (each morning, each evening) reminding participants to use the app. This may have encouraged an association between time of day and app use. In line with this, appreciation of these prompts emerged as another subtheme, consistent with prior research [19,21,24,27]. Outside of the mHealth app literature, it is encouraging that app use became routine for these study participants considering the merits of practicing self-regulation skills regularly. When a person uses strategies like those provided in the JoyPop app when they are not necessarily distressed, the strategies are well-practiced and ready to be used when distress does occur, potentially obviating the need for later reactive strategies [58]. It should be noted, however, that a few participants commented that if they were too busy with school life, they would use the app less, suggesting that the power of routine may not be strong enough to promote app use during especially stressful times for students.

Other facilitators of app use endorsed by participants were the self-monitoring and expressive opportunities provided by the JoyPop app. Consistent with this, the features that most clearly support these functions were among the most used and most helpful features according to participants, such as the Rate My Mood and Journal features. Prior studies have consistently indicated that self-monitoring and tracking of one’s mood is a highly desired feature of mental health–related apps [18,21,24,27], and our findings are no exception. The importance of self-monitoring to participants was further demonstrated by their desire for more tracking abilities within the app, such as being able to look back at one’s mood ratings or see charts and graphs of mood changes over time. This is consistent with prior research indicating that enhanced tracking ability is one of the most common app features requested by users [13,18]. Self-monitoring and the ability to identify patterns in emotional responding is integral to many forms of psychotherapy [59], and it seems to be an aspect that participants also value and desire to engage in further.

Related to the self-monitoring and expressive opportunities that facilitated app use were the perceived outcomes described by participants of checking in with oneself, increased awareness, and emotional control. The JoyPop app appeared to serve as a signal to participants to pause their day to check in on how they were doing and assess how they might improve their mood if needed. By engaging in this self-monitoring and expression of emotion, the participants seemed to gain awareness and understanding of their emotions to better regulate them. Specifically, they indicated that the Journal and Rate My Mood features helped them learn to label and communicate their feelings, while the Breathing Exercises also provided a more direct form of emotion regulation. These findings are in line with general theories of self-regulation, which emphasize a connection between self-reflection, understanding, and regulation [31,60]. Further, the participants’ perceptions of improved emotion regulation were mirrored by our quantitative results, which spoke to a reduction in emotion dysregulation with each additional day of app use over 4 weeks [9]. As such, there is encouraging convergence of quantitative and qualitative findings.

Another outcome of app use was that participants found it acted as a helpful distraction. Both SquareMoves and the Journal feature were mentioned within this subtheme, such that participants reported they felt these activities helped them take their mind off negative emotions and thoughts and focus on something else. Interestingly, in our tally of feature use and helpfulness, SquareMoves was indicated as one of the most used features; however, it was rarely mentioned as the most helpful feature. This finding suggests that it was the entertainment or distraction value of the feature that fostered continued use. Models of technology acceptance highlight entertainment factors as an important motive to using mHealth apps [21,57], such that “gamification,” or including game-like design elements, can be useful to increase user engagement with apps [61]. As such, the helpfulness of SquareMoves may have been masked by its entertainment value; however, distraction can still function as a valid form of emotion regulation, particularly in situations when emotion is overwhelming [62]. In essence, some students may have used the app for fun or as a distraction, which can still indirectly support emotion regulation.

In terms of constructive criticism, participants described barriers to app use and recommendations for app improvements. They desired enhanced functionality of certain features, such as the Journal (more prompts, the ability to edit entries) and the Rate My Mood feature (greater variety of mood states). They also desired more features overall and were forthcoming with suggestions about features that should be added. This is in line with prior research on mHealth apps, which suggests that app users desire a sufficient number and diverse array of features that are also flexible enough to accommodate various needs [18,21,25,27]. Indeed, reviews of mood-monitoring apps on popular app download sites often cite a lack of features as a primary reason for user dissatisfaction with apps [27]. Further, participants in our study recommended that features should be more personalized. Similarly, previous studies have found that users want customization and some degree of control over app features [21,25,27,37,63]. Finally, captured under the subtheme of increased stress, 2 participants commented that the appearance of the app contained too many buttons, distracting colors, and repetitive appearance of quotes, which rendered the app less helpful. Appearance and design tend to be major themes of user feedback on mHealth apps [18,21,24,25,27,29,37,64], and users may not use an app that is not user friendly even if they can see its benefit [24]. Therefore, these comments represent important feedback. However, the relatively few comments of this nature suggest that the general look-and-feel of the JoyPop app was acceptable to most participants.

Certain features did not appear instrumental to the uptake and perceived helpfulness of the JoyPop app. These included the Circle of Trust and Art features, which were either rarely mentioned or mentioned as the least used and least helpful features. With respect to the Circle of Trust feature, this finding is somewhat surprising, as having some aspect of social support and feeling part of a community have been cited as important to app users in prior research [18,25,37,63]. The social support in these studies, however, pertained to the ability to share app data with other users or in an online forum, including with close others or anonymously with other app users [21,24,25,37]. This is different from the social support included in the JoyPop app, which allows users to input and contact family, peers, and other support persons in times of need. Peng et al [21] noted that some participants felt that seeing others using the app through data sharing would motivate them to use the app. Similarly, our results indicated some participant desire to enhance the social aspect of the app in a similar fashion, such as being able to communicate with others directly in the app so that users might hold each other accountable. With respect to the Art feature, this activity may have been relatively unused due to the inability to save drawings, which was one drawback expressed by participants. However, ongoing evaluation among more diverse groups would be warranted, as it is possible that our student sample, which consisted mainly of students pursuing health or science degrees, may have been less interested in or drawn to the Art feature.

### Strengths, Limitations, and Future Directions

This study had many strengths. Obtaining user input on experiences with the JoyPop app and their perspectives on its utility aligns with recommendations of the value of user involvement in the design of digital health interventions [64-67]. With respect to data collection and analysis, the authors who conducted the analyses were independent from the study conceptualization and data collection to avoid interpretation bias. Additionally, the questions chosen for the interview guide served several functions that are important to comprehensive app evaluations, including discovering what is important to users for long-term uptake of the app, which remains an ongoing issue for many app endeavors [26].

A limitation to the current study is that participants were predominantly female, which reflects the composition of the larger sample of participants who used the app from which this subsample was drawn [9]. Certainly, there are potential gender differences in youths’ preferences for and use of mental health-based apps [37]. Male students are also relatively underrepresented in studies of mental health interventions for university students in general [68]. Given that our sample consisted of university students in their late teens, additional research examining younger youths’ perspectives on the app will be important before wider implementation occurs.

A further limitation is the variation in the number of participants who attended each interview session, which ranged from 1 to 5. A larger group may have resulted in some participants sharing more than others, which may have created differences in how much certain perspectives were amplified. In addition, participants may have been less willing to share negative feedback about the JoyPop app in the presence of the interviewers; however, certain questions were meant to specifically elicit negative or constructive feedback (eg, least used/least helpful features, barriers to use, recommendations for improvement), which should have encouraged participants to provide honest feedback about the app.

Continuing to seek and incorporate feedback from users into evolving versions of the JoyPop app is an important future direction. Understanding user perspectives on the utility, features, and design of the app will aid in ensuring its continued use and engagement [64-67]. Consistent with participant recommendations to add more features and enhance existing features, a new SleepEase feature offering relaxation exercises and tips for preparing the body for sleep has been added to the latest version [69]. In addition, more journal prompts have been added, and users are now informed during the orientation to the app about methods they can use to save their Art drawings (eg, by screenshotting and saving them directly to their devices). Consideration of participants’ suggestions for increased personalization (eg, location-based and culture-related customization) of the JoyPop app will be important in future updates; for instance, the latest iteration of the JoyPop app includes a French-language version.

Although the JoyPop app was designed specifically to draw attention toward positive mood states, consistent with consumer desire for positivity in mHealth apps [20,40], it is important to consider participant requests for opportunities to rate and track a range of emotions over time. Should additional mood ratings and tracking abilities be added to subsequent versions of the JoyPop app, ongoing evaluation of the consequences of this change would be warranted to ensure that no unintended negative consequences emerge (eg, worse mood after reviewing past mood ratings). Following recommendations in two recent reviews, additional research exploring the economic impact of app-based interventions is an important next step [70,71]. For instance, it will be important to examine the economic impact, including cost-effectiveness and cost utility, of integrating an intervention such as the JoyPop app into school or mental health settings for youth. Finally, due to ongoing disruptions related to the COVID-19 pandemic, including prolonged changes to education, mental health service delivery, and social practices, optimizing digital approaches to service delivery (eg, mHealth apps) is a growing global priority [72,73]. As such, exploring whether the JoyPop app can aid in buffering against the negative effects of stress brought on by the ongoing global pandemic, or how users’ perspectives on the app may vary within the context of the pandemic, is warranted.

### Conclusion

In sum, this qualitative study of users’ experiences with the JoyPop app provides insight into aspects of the app that motivated and benefitted users, as well as actions that can be taken to improve user experiences and promote longer-term uptake. Students in our study demonstrated a willingness to engage with the app and incorporate it into their routine, while valuing the ability to self-monitor, express emotion, and engage in distraction. They preferred and used some features over others and were forthcoming with various suggestions for improvement. Their feedback underscores the value of considering user input in the continual development and evolution of apps, and it will be instrumental in ensuring the JoyPop app meets the needs of its users.

## Appendix

Multimedia Appendix 1 Interview guide.

## Abbreviations

mHealth: mobile health
