# Supplementary material for: User Perspectives on a Resilience-Building App (JoyPop): Qualitative Study
Source: JMIR Mhealth Uhealth. 2021 Jul 8;9(7):e28677. doi: 10.2196/28677 (PMC8299348; doi:10.2196/28677)
Supplement: Multimedia Appendix 1 [file mhealth_v9i7e28677_app1.pdf]

Multimedia Appendix 1: Interview Guide

1. Approximately how often did you use the app?

2. Have you ever used anything like this before and, if yes, please describe it?

3. What is your understanding of the purpose of the app?

4. What were your expectations about what the app would do?
    a. How did it meet your expectations?

5. What were your expectations about how the app would affect you?
    b. How did it meet your expectations?

6. Overall, tell me about your experience using the app.

7. Which features did you use most often and why?

8. Which features did you like the most and why?

9. Which features did you find the most helpful and why?

10. Which features did you use the least often and why?

11. Which features did you like the least and why?

12. Which features did you find the least helpful and why?

13. Did anything interfere with you using the app?

14. What would make you more likely to use the app?

15. What would ensure your continued use of the app?

16. Do you have any recommendations or suggestions to improve the app?

17. Do you have any recommendations or suggestions to increase the likelihood of someone else using the app in the future?

18. Is there anything else you would like to share about your experience with the app?
